# Estimation of Heating Temperature for Fire-Damaged Concrete Structures Using Adaptive Neuro-Fuzzy Inference System

**DOI:** 10.3390/ma12233964

**Published:** 2019-11-29

**Authors:** Hyun Kang, Hae-Chang Cho, Seung-Ho Choi, Inwook Heo, Heung-Youl Kim, Kang Su Kim

**Affiliations:** 1 Korea Institute of Civil Engineering & Building Technology (KICT) 182-64 Mado-ro, Mado-myeon, Hwaseong 18544, Gyeonggi Province, Korea; kanghty@kict.re.kr (H.K.); hykim@kict.re.kr (H.-Y.K.); 2 Department of Architectural Engineering, University of Seoul 163 Seoulsiripdae-ro, Dongdaemun-gu, Seoul 02504, Korea; chang41@uos.ac.kr (H.-C.C.); ssarmilmil@gmail.com (S.-H.C.); inwookheo@gmail.com (I.H.)

**Keywords:** ANFIS, concrete, fire, fuzzy, heating temperature, membership function

## Abstract

The structural performance of concrete structures subjected to fire is greatly influenced by the heating temperature. Therefore, an accurate estimation of the heating temperature is of vital importance for deriving a reasonable diagnosis and assessment of fire-damaged concrete structures. In current practice, various heating temperature estimation methods are used, however, each of these estimation methods has limitations in accuracy and faces disadvantages that depend on evaluators’ empirical judgments in the process of deriving diagnostic results from measured data. Therefore, in this study, a concrete heating test and a non-destructive test were carried out to estimate the heating temperatures of fire-damaged concrete, and a heating temperature estimation method using an adaptive neuro-fuzzy inference system (ANFIS) algorithm was proposed based on the results. A total of 73 datasets were randomly extracted from a total of 87 concrete heating test results and we used them in the data training process of the ANFIS algorithm; the remaining 14 datasets were used for verification. The proposed ANFIS algorithm model provided an accurate estimation of heating temperature.

## 1. Introduction

**Figure 1:**
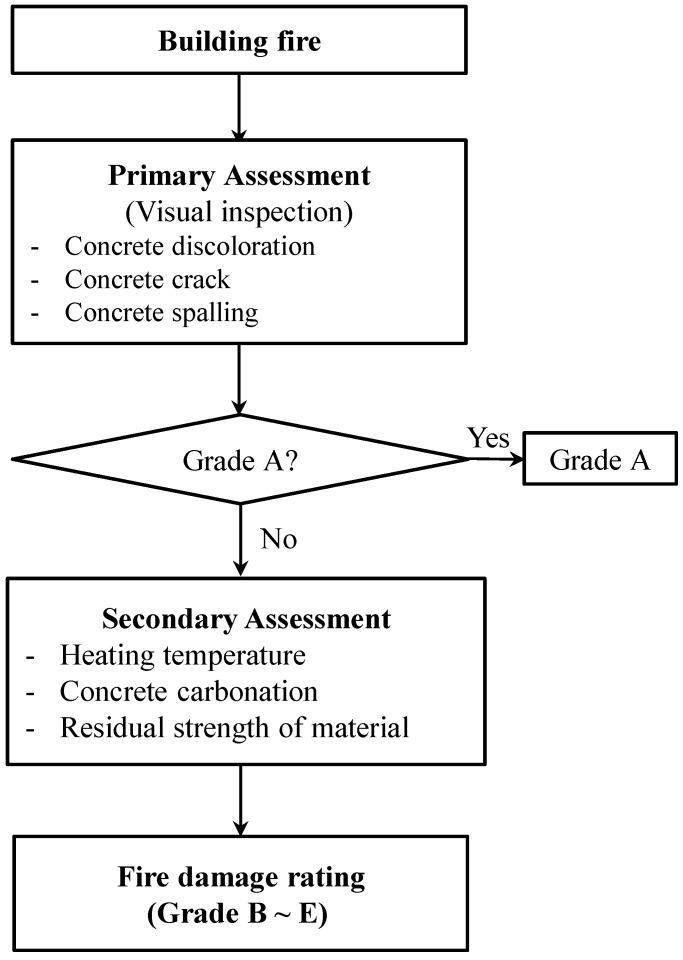
Existing diagnosis process for fire-damaged structures [5].

Inspection methods for the diagnosis and maintenance of buildings are very diverse. In recent years, methods for quantitatively measuring deformation of structures using camera equipment have been proposed [1,2,3]. However, the fire damaged buildings exhibit different behavior patterns from the aging or deterioration of buildings depending on the fire temperature. For this reason, a diagnostic method for fire damaged buildings is required. Concrete is a composite material made from a mixture of cement, aggregate, and water. When exposed to high temperatures, concrete expands in ways that depend on the thermal properties of each component, which may result in concrete cracks and spalling [4,5,6,7]. This phenomenon, in turn, drastically decreases the compressive and tensile strengths and durability of concrete. Furthermore, severe performance degradation may lead to failures in concrete structural members or even the collapse of the structure. Therefore, in the event of a fire, it is very important to take the appropriate follow-up measures needed for an accurate diagnosis and assessment of the fire-damaged structure. The Architectural Institute of Japan [8] requires that if a fire breaks out in a building, the diagnosis and assessment of the fire-damaged building should be performed based on the procedures shown in Figure 1. For instance, if the visual inspection determines that the damage is insignificant, the fire-damage rating is Grade A; otherwise, the damage will be rated by means of a secondary assessment. Table 1 compares the rating tables currently used in Korea and Japan with those used in the United States (US) [9,10,11], and their grade description of fire damage rating was not significantly different. However, it seems that the US grade table is described in more detail.

The heating temperature changes the thermal properties of concrete, which subsequently leads to a significant change in the behavior of the structural members [12]. Therefore, in the diagnosis and assessment of fire-damaged structures, accurate estimations of the heating temperatures experienced by concrete are critical in assessing the residual structural performance. The simplest way to estimate the heating temperature of concrete is to inspect the discoloration of the concrete surface and spalling with the naked eye. However, this method poses a difficulty in quantitatively estimating heating temperatures accurately and has a limitation because of its heavy dependence on the experience of a person responsible for the diagnosis [13]. Other heating temperature estimation methods include the ultraviolet (UV) spectrum method [14], ultrasonic spectroscopy method [15], X-ray diffraction method [16], and measurement of the carbon dioxide re-absorption amount method [17]. However, the range of heating temperatures that can be estimated by each method is limited, and the results may differ significantly depending on the type of chemical admixtures used in the concrete mix. In addition, there is a problem in that the whole process of deriving assessment results from the measured diagnostic data is greatly dependent on the evaluator’s empirical judgment.

The method of estimating heating temperature using statistical learning theory [18] with ultrasonic pulse velocity, splitting tensile strength of concrete and moisture absorption was proposed. In a fire damaged structure, however, the water absorption parameter of the concrete can be significantly different from the actual situation due to the water spray for extinguishing the fire. Therefore, it is difficult to derive actual heating temperature using the existing temperature estimation methods.

This study proposed a heating temperature estimation method using an adaptive neuro-fuzzy inference system (ANFIS) to overcome the problems of existing heating temperature estimation methods. The ANFIS is a system that applies a training algorithm to the fuzzy theory and can solve complex problems, such as heating temperature estimation, which is hard to do using structural or numerical analysis. Furthermore, it has the advantage of providing a quantitative output by means of comprehensive considerations of the correlations between various input parameters [19]. Thus, it can minimize the evaluator’s subjective judgment in the entire process of deriving assessment results from the measured diagnostic data. In this study, the heating test and non-destructive test were conducted on concrete specimens, and the ANFIS algorithm [20,21]. How to estimate heating temperatures was then developed based on the test results. In addition, the accuracy of the proposed model was verified by comparing the temperature of the furnace measured in the concrete heating test with the heating temperature estimated using the proposed ANFIS algorithm.

## 2. Concrete Heating Test

### 2.1. Test Program

Table 2 summarizes the compressive strengths of concrete used in the concrete heating test, the number of specimens for each compressive strength, and target temperatures. The test specimens included six types in terms of compressive strengths, from normal-strength concrete (24 MPa) to high-strength concrete (80 MPa), and a total of seven target temperatures, ranging from 25 °C to 800 °C were taken into consideration. The target temperature was limited to 800 °C due to the safety concerns on the furnace and researcher as well. In addition, 21 specimens per compressive strength were fabricated, and three specimens were tested for each target temperature and concrete compressive strength. However, specimens with a design compressive strength of 80 MPa, which underwent spalling at a relatively low target temperature of 400 °C, were excluded from the tests with target temperatures of 600 °C, 700 °C, and 800 °C.

**Figure 2:**
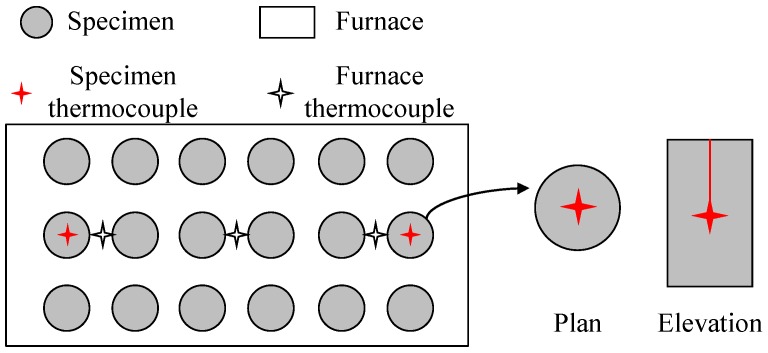
Location of thermocouples.

Figure 2 shows the specimen layouts and thermocouple installation locations inside the furnace. In order to measure the temperatures during the test, thermocouples were installed in the furnace and specimens. The thermocouples (TSTEC, Seoul, Korea) installed in the specimens were placed in the center of the cross-section in order to check if the temperature inside the specimen reached the target temperature.

### 2.2. Heating Test Results

**Figure 3:**
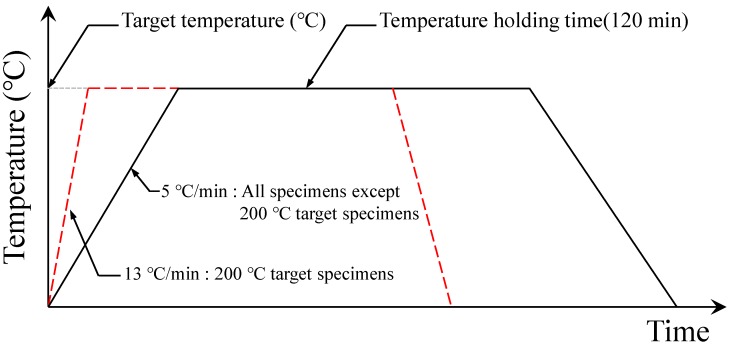
Heating rate of concrete fire test.

**Figure 4:**
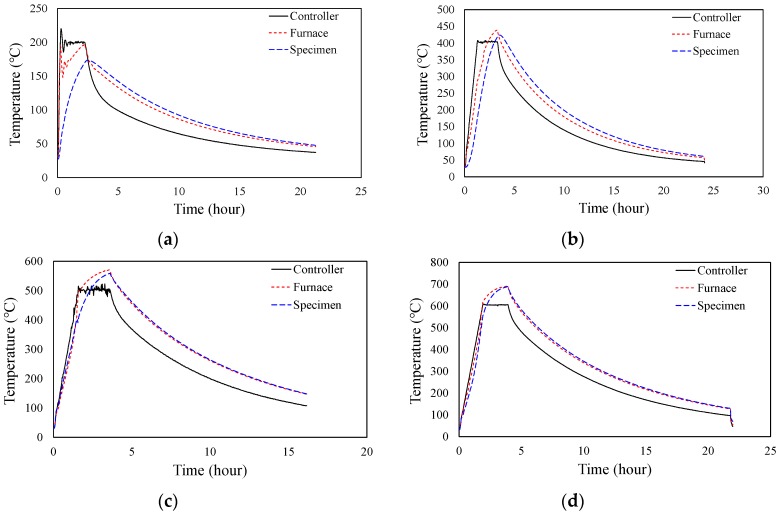

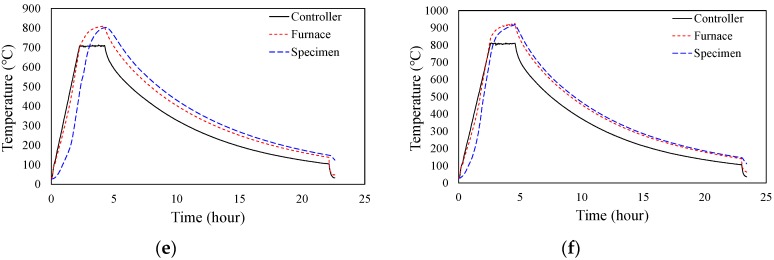
Temperature records of furnace and specimen. (**a**) Target temperature: 200 °C; (**b**) Target temperature: 400 °C; (**c**) Target temperature: 500 °C; (**d**) Target temperature: 600 °C; (**e**) Target temperature: 700 °C; (**f**) Target temperature: 800 °C.

Figure 3 shows the heating rate of the concrete fire test. A heating rate of 13 °C/min was applied in the test with a target temperature of 200 °C. The specimen reached the target temperature at about 15 min after heating. Later, it maintained the target temperature for 120 min and was then naturally cooled to room temperature (20 °C) upon completion of the test. Figure 4 shows the average internal temperature of the furnace, the average internal temperature of the specimen, and the furnace controller temperature measured during the heating test. All of the specimens cooled for more than 20 h, but the specimens with a target temperature of 500 °C were measured for 16 h only due to unexpected interruption of data logger. As identified in Figure 4a, a slight difference between the average internal temperature of the furnace and that of the specimen was observed in the test with a target temperature of 200 °C. This suggests that, since it takes a certain time for the temperature on the surface of the specimen to be transmitted inside (i.e., thermal conduction), and the initially set heating rate is rather large, a difference arises between the average internal temperature of the furnace and that of the specimen during heating. In other words, the temperature difference between the specimen and the furnace increases as the target temperature rises. Therefore, in the test with a target temperature of over 400 °C, the heating rate was lowered to 5 °C/min. As a result, the average internal temperatures of the furnace and of the specimen were similar in all the tests except for the one with a target temperature of 200 °C as shown in Figure 4b–f.

According to previous research [22,23,24,25], concrete spalling occurs at temperatures ranging from 250 to 350 °C, and high-strength concrete is more vulnerable to spalling than is normal-strength concrete. Table 3 shows a summary of the temperatures of specimens that underwent spalling during the concrete heating test and the internal average temperature of the furnace at spalling. The point of time for spalling was set as the time when the spalling sound occurred during the test. In the test with a target temperature of 200 °C, spalling did not occur, and the spalling was observed mostly in the specimens with the highest concrete compressive strengths. The average temperature of the specimen and that of the furnace at spalling were 270 and 433 °C, respectively. Similar to previous research [22,23,24,25] results, high-strength concrete (56.9, 77.1 MPa) specimens underwent spalling at a relatively low average furnace temperature.

## 3. Non-Destructive Test

In order to develop the ANFIS algorithm for estimating the heating temperature of concrete, factors which are closely related to the heating temperature should be configured as input parameters. Previous research [14,15,16,17,18] has reported that when concrete is subjected to temperature loads, the ultrasonic pulse velocity, the color components of the concrete surface, and the light reflectance and wavelength of light, change. Therefore, in this study, an ultrasonic pulse velocity test and colorimetric analysis were carried out, and these were used as input parameters of the ANFIS algorithm.

### 3.1. Ultrasonic Pulse Velocity Test

**Figure 5:**
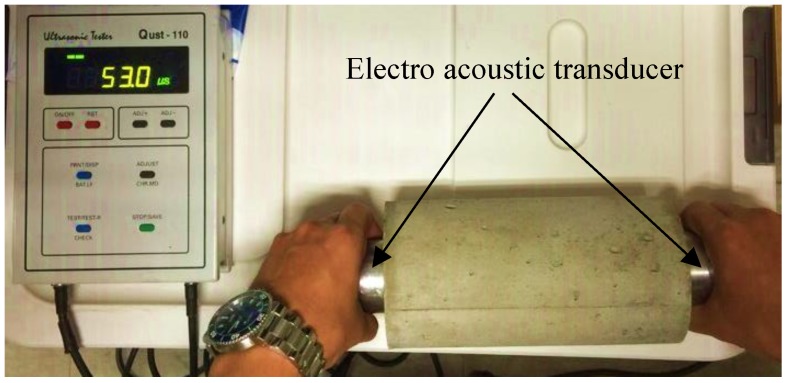
Ultrasonic pulse velocity test.

The ultrasonic pulse velocity test measures the rate at which ultrasonic waves are transmitted between electro-acoustic transducers after the transducers, composed of the receiver and the transmitter, come into contact with both sides of the concrete specimen, as shown in Figure 5 [26]. Unlike undamaged concrete with an airtight internal structure, fire-damaged concrete suffers from micro-pores and cracks because of the decomposition of calcium-silicate-hydrate (C–S–H), which is the main component of cement paste, in the process of dehydration. This decreases the ultrasonic pulse velocity. In this study, ultrasonic pulse velocities were measured on specimens that were naturally cooled after the heating test and non-heated specimens. However, since specimens with thermocouples installed or those with surface damages may provide inaccurate values, they were excluded from the measurements. Table 4 summarizes the ultrasonic pulse velocity measurement results of specimens after the heating test. The specimens with the same target temperature exhibited similar ultrasonic pulse velocities, which decreased with increasing fire exposure temperatures. On the other hand, there was no significant tendency of the ultrasonic pulse velocity that depended on the concrete compressive strength.

### 3.2. Colorimetric Analysis

**Figure 6:**
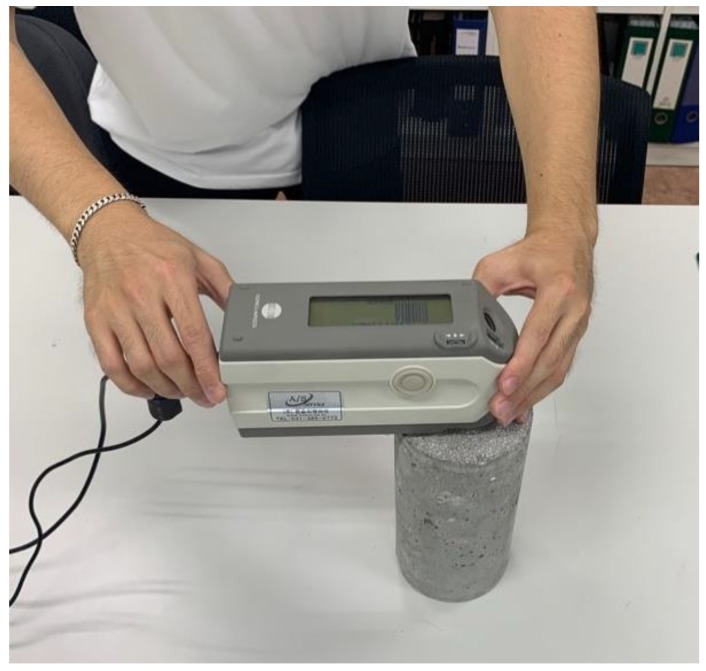
Spectrophotometer.

**Figure 7:**
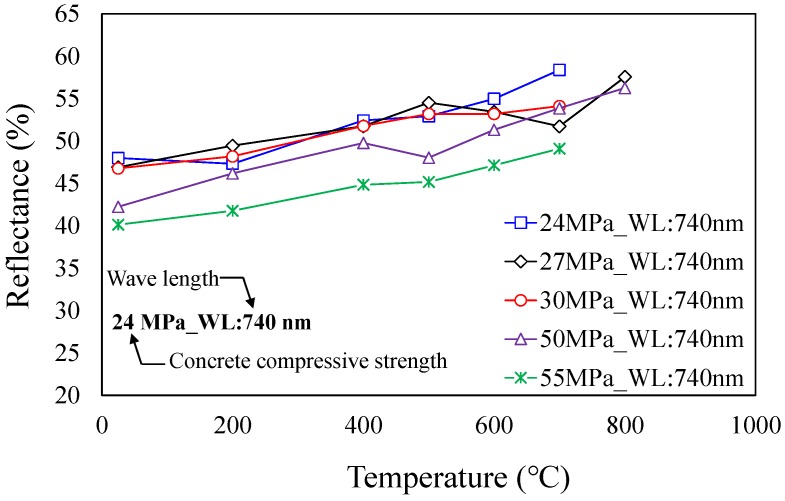
Reflectance of concrete surface.

In general, when concrete is exposed to high temperatures, soot forms on the surface of the concrete at heating temperatures below 300 °C, the concrete surface turns pink, light gray, and light yellow at heating temperatures over 300 °C, and concrete melts at very high heating temperatures over 1200 °C [11,27,28]. In the diagnosis of buildings damaged by fire exposure, concrete discoloration on the surface is examined by visual inspection, and the range of heating temperature is indirectly estimated as shown in Table 5. However, this diagnostic method has disadvantages in that the subjective judgment of the person responsible for the diagnosis is very likely to intrude into the diagnostic process, because it is difficult to accurately identify the discoloration of concrete surfaces, and the estimation range of heating temperatures according to the discoloration status is extremely wide. Therefore, in this study, a colorimetric analysis was performed to quantitatively consider the discoloration characteristics of fire-damaged concrete. The colorimetric analysis is a way to analyze the light reflectance, the wavelength of light, and the color components of the fire-damaged concrete surface by using a spectrophotometer, shown in Figure 6. The results of the colorimetric analysis using the spectrophotometer are shown in Figure 7, where the wavelength of light and color components of the concrete surface, from which significant tendencies were not measured, were excluded from the analysis. The reflectance of the concrete surface was found to increase in proportion to the heating temperature, and high-strength concrete ($f_{ck}\geq 50\;\mathrm{MPa}$) exhibited reflectance much lower than that of normal-strength concrete ($f_{ck}<50\;\mathrm{MPa}$).

## 4. Adaptive Neuro-Fuzzy Inference System

The results of the ultrasonic pulse velocity test and colorimetric analysis on fire-damaged concrete showed that there are certain tendencies according to the heating temperature. Based on the results, the design compressive strength of concrete, ultrasonic pulse velocity, and reflectance of concrete surface were set as the ANFIS input parameters. It should be noted that in the actual fire-damaged structure, the ultrasonic pulse velocity and reflectance of concrete can be obtained by means of diagnosis, but it is impossible to identify the compressive strength of the concrete of the structural member before fire exposure. In such cases, the evaluator should do a diagnosis using information about the design compressive strength of concrete. Therefore, the design compressive strength of concrete rather than the actual compressive strength of concrete was set as the ANFIS input parameter in this study.

The fuzzy theory, which serves as the basis of ANFIS, converts data that is difficult to digitize, such as linguistic variables, into a quantitative membership function to process data [19]. For example, in the general proposition, the case in which the element is a member of a crisp set is indicated as ‘True (1)’, and the case in which it is not an element of the set is indicated as ‘False (0)’. On the other hand, in a fuzzy set, as the crisp value undergoes fuzzification via membership functions, its degree of membership in the fuzzy set can be indicated as a possibility quantified as a number between 0 and 1 [20,29,30]. Therefore, it is possible to digitize various possibilities that might occur in a particular event in order to make a judgment. However, since the reliability of the results of a methodology applying the fuzzy theory may differ significantly depending on the configuration method of fuzzy sets and rules, the latter should be defined by referring to reliable data and codes [31], and the fuzzy sets and rules should be optimized by means of iterative calculations in order to improve the accuracy of the output. The ANFIS is based on Sugeno fuzzy inference [32,33], and can be optimized without directly modifying fuzzy sets and rules by means of a data-training system applied with back-propagation algorithms [20].

**Figure 8:**
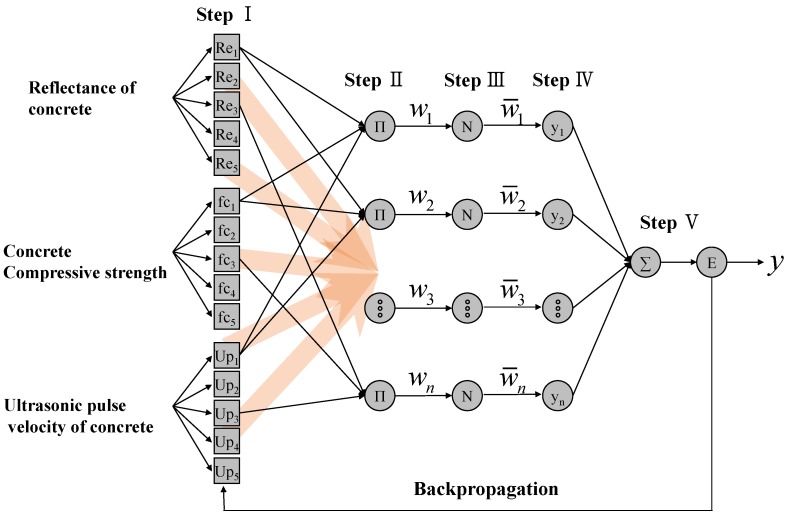
Adaptive neuro-fuzzy inference system ANFIS algorithm for estimation of heating temperature.

**Figure 9:**
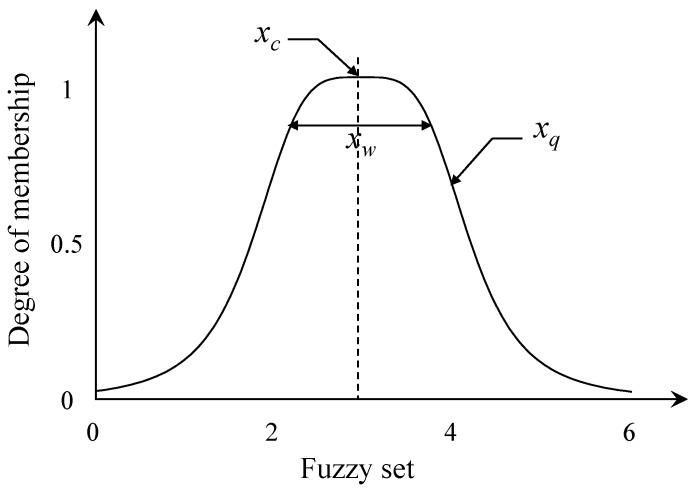
Bell-shaped fuzzy set.

**Figure 10:**
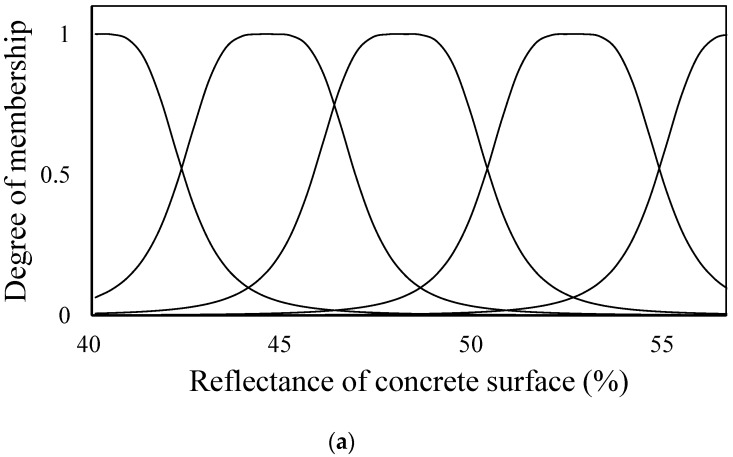

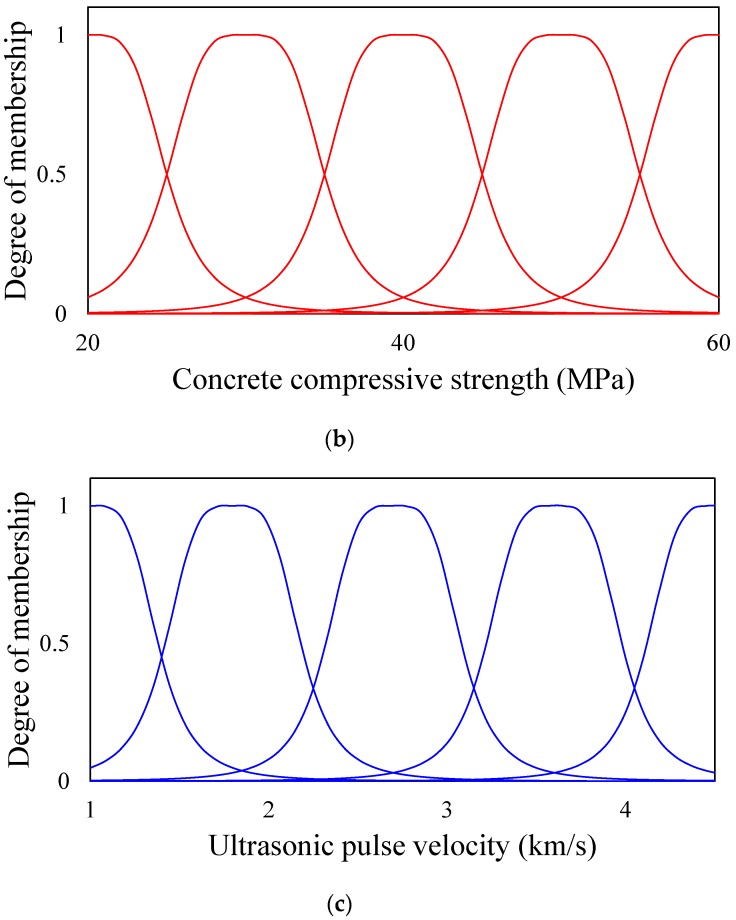
Fuzzy sets of input parameters before training. (**a**) Reflectance of concrete surface; (**b**) Concrete compressive strength; (**c**) Ultrasonic pulse velocity.

In this study, a 5-step ANFIS algorithm for estimating heating temperatures was configured, as shown in Figure 8. The ultrasonic pulse velocity, reflectance of concrete, and design compressive strength of concrete on the left side of Figure 8 are the input parameters of the ANFIS algorithm, using the results of the non-destructive test on the concrete heating test specimens described in Table 2. Step I in the ANFIS algorithm is the fuzzification process of the input parameters. In this process, a bell-shaped function, as shown in Figure 9, was applied as a fuzzy set for each input parameter. Therefore, the membership function (*w*) can be represented as below.

(1) $$bell\left(x_{i}^{j};x_{c},x_{w},x_{q}\right)=\frac{1}{1+{\left|\frac{x_{i}^{j}-x_{c}}{x_{w}}\right|}^{2x_{q}}}$$

where $x_{i}^{j}$ is the input parameter, and $x_{c}$, $x_{w}$, and $x_{q}$ are the center, width, and shape parameter of the fuzzy set, respectively. In the ANFIS algorithm, $x_{c}$, $x_{w}$, and $x_{q}$ are defined as premise parameters. Based on the test results, this study assumes the initial premise parameters for each input parameter (ultrasonic pulse velocity, reflectance of concrete, and design compressive strength of concrete), and five fuzzy sets are configured for each input parameter, as shown in Figure 10.

In Step II, the number of possible cases for each fuzzified input parameter is taken into consideration to configure the fuzzy rules. In this study, because each of the three input parameters has five fuzzy sets, a total of fuzzy rules ($fr_{n},i=1,2,\ldots ,125$) are configured, as follows:

(2) $$\begin{array}{l} fr_{1}=a_{1}R_{e}{}_{1}^{1}+b_{1}f_{c}{}_{1}^{2}+c_{1}U_{lt}{}_{1}^{3}+d_{1}\\ fr_{2}=a_{2}R_{e}{}_{2}^{1}+b_{2}f_{c}{}_{2}^{2}+c_{2}U_{lt}{}_{2}^{3}+d_{2}\\ fr_{3}=a_{3}R_{e}{}_{3}^{1}+b_{3}f_{c}{}_{3}^{2}+c_{3}U_{lt}{}_{3}^{3}+d_{3}\\ \begin{array}{cccc} & & \begin{array}{ccc} \begin{array}{ccc} & & \vdots \end{array} & & \end{array} & \end{array}\\ fr_{125}=a_{125}R_{e}{}_{125}^{1}+b_{125}f_{c}{}_{125}^{2}+c_{125}U_{lt}{}_{125}^{3}+d_{125} \end{array}$$

where $R_{ei}^{1}$, $f_{ci}^{2}$, and $U_{lti}^{3}$ are the input parameters representing the reflectance of concrete surface, the design compressive strength of concrete, and the ultra-pulse velocity of the concrete, respectively. In addition, $a_{i}$, $b_{i}$, $c_{i}$, and $d_{i}$ are the constant terms of linear functions calculated using the least-square estimation and defined as consequent parameters in the ANFIS.

In Step III, the firing strength of fuzzy rules configured in Step II is calculated and normalized for defuzzification. The firing strength ($w_{i}$) is calculated using T-norm methods [20] as below.

(3) $$\begin{array}{l} w_{i}=T\left(\mu_{Re_{k}},\mu_{fc_{k}},\mu_{Ult_{k}}\right)\\ i=1,2,\ldots ,125\\ k=1,2,3,4,5 \end{array}$$

where μRek, $\mu_{fc_{k}}$, and $\mu_{Ult_{k}}$ are the membership degrees of the fuzzy set for the reflectance of the concrete surface, the design compressive strength of concrete, and the ultrasonic pulse velocity of the concrete, respectively. If the firing strength calculated using Equation (3) is normalized, that strength (${\bar{w}}_{i}$) can be represented as follows.

(4) $${\bar{w}}_{i}=\frac{w_{i}}{\sum_{i=1}^{125}w_{i}}$$

In Step IV, the normalized firing strength (${\bar{w}}_{i}$) calculated in Step III is reflected in the fuzzy rule to normalize it, and the normalized fuzzy rule ($y_{i}$) is calculated as below.

(5) $$y_{i}={\bar{w}}_{i}fr_{i}={\bar{w}}_{i}\left(a_{i}R_{e}+b_{i}f_{c}+c_{i}U_{lt}+d_{i}\right)$$

In Step V, the normalized fuzzy rule ($y_{i}$) calculated by means of Equation (5) is defuzzified, and the heating temperature of the concrete ($y_{ANFIS}$) is calculated using the centroid method [34], as follows:

(6) $$y_{ANFIS}=\sum_{i=1}^{125}y_{i}=\sum_{i=1}^{125}{\bar{w}}_{i}fr_{i}$$

The ANFIS algorithm minimizes the number of errors by means of the least-square estimation and back-propagation algorithm [20] to increase the accuracy of the resulting values. The least-square estimation is used to calculate $a_{i}$, $b_{i}$, $c_{i}$, and $d_{i}$, which are consequent parameters in Equation (2), and the sum of the squares of the errors (E(θ)) between the heating temperature calculated by means of the ANFIS and the heating temperature measured in the actual test can be calculated as shown below.

(7) $$\begin{array}{l} E\left(\theta \right)=\sum_{i=1}^{m}{\left(y_{rp_{i}}-i_{i}^{T}\theta_{i}\right)}^{2}=e^{T}e={\left(Y_{rp}-I\Theta \right)}^{T}\left(Y_{rp}-I\Theta \right)\\ I=\left[\begin{array}{l} \begin{array}{ccc} \bar{w_{1}}R_{e1}\bar{w_{1}}f_{c1}\bar{w_{1}}U_{lt1} & \cdots & \bar{w_{125}}R_{e1}\bar{w_{125}}f_{c1}\bar{w_{125}}U_{lt1}\\ \bar{w_{1}}R_{e2}\bar{w_{1}}f_{c2}\bar{w_{1}}U_{lt2} & \cdots & \bar{w_{125}}R_{e2}\bar{w_{125}}f_{c2}\bar{w_{125}}U_{lt2}\\ & \vdots & \end{array}\\ \begin{array}{ccc} \bar{w_{1}}R_{en}\bar{w_{1}}f_{cn}\bar{w_{1}}U_{ltn} & \cdots & \bar{w_{125}}R_{en}\bar{w_{125}}f_{cn}\bar{w_{125}}U_{ltn} \end{array} \end{array}\right]\\ \Theta =\left[\begin{array}{cccc} a_{1} & b_{1} & c_{1} & d_{1} \end{array}\begin{array}{cccc} \cdots & a_{125} & \cdots & d_{125} \end{array}\right] \end{array}$$

where m is the number of data, $Y_{rp}$ is the heating temperature test value, I is the input parameter, and Θ are the consequent parameters. The consequent parameter (Θ) when E(θ) becomes the minimum value, at which the rate of change of E(θ) is zero (i.e., $E\left(\theta \right)/\partial \theta =2I^{T}I\Theta -2I^{T}Y_{rp}=0$), can be calculated as follows.

(8) $$\Theta ={\left(I^{T}I\right)}^{-1}I^{T}Y_{rp}$$

The back-propagation algorithm is applied to optimize the premise parameters $x_{c}$, $x_{w}$, and $x_{q}$ of Equation (1). The consequent parameters (Θ) determined through the least squares is reflected in Equation (6) to calculate the heating temperature ($y_{ANFIS}$), and the error ($E_{p}$) is recalculated by comparing it with the heating temperature test value ($y_{rp}$), as follows:

(9) $$E_{p}=\sum_{i=1}^{m}{\left(y_{rp_{i}}-y_{ANFIS_{i}}\right)}^{2}$$

From Equation (9), the error increment ($\Delta x_{cwq}$) can be calculated as follows.

(10) $$\Delta x_{cwq}=-\frac{\partial E_{p}}{\partial x_{cwq}}$$

where $x_{cwq}$ are the premise parameters ($x_{c}$, $x_{w}$, $x_{q}$). The error increment ($\Delta x_{cwq}$) is reflected in the $t^{\prime }+1$-th premise parameters along with the learning rate (η), as follows:

(11) $$x_{cwq}\left(t’+1\right)=x_{cwq}\left(t’\right)-\frac{\eta }{m}\Delta x_{cwq}$$

**Figure 11:**
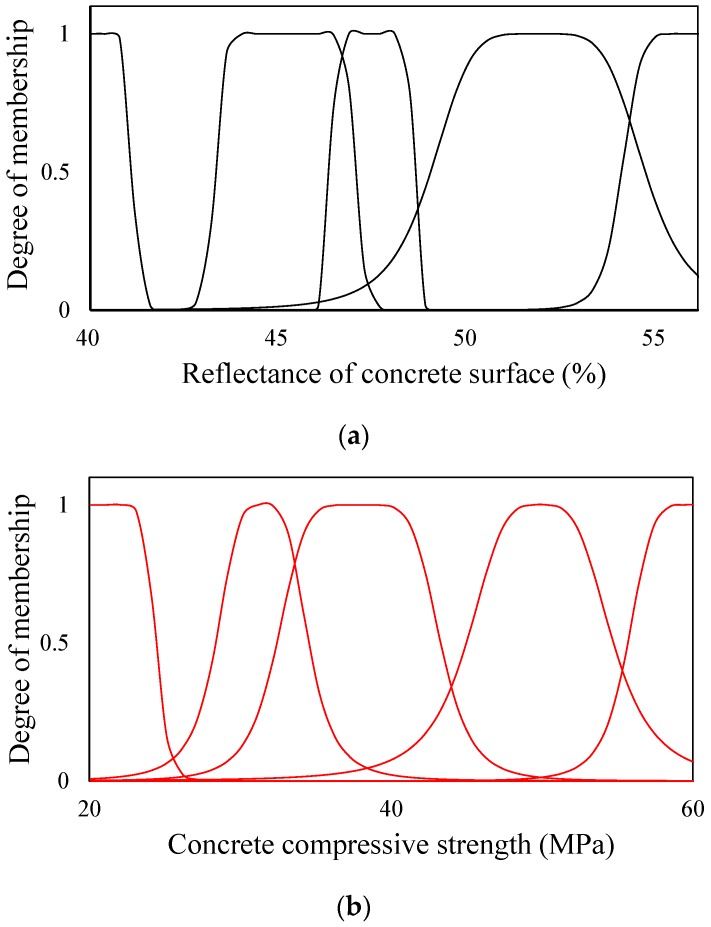

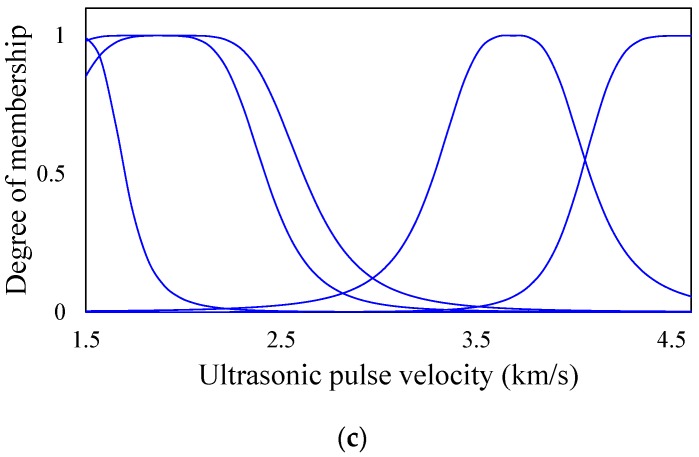
Fuzzy sets of input parameters after training. (**a**) Reflectance of concrete surface; (**b**) Concrete compressive strength; (**c**) Ultrasonic pulse velocity.

The fuzzy sets are then redefined using the updated conditional factors, and the fuzzy rules are reconfigured to recalculate the consequent parameters. That is, the calculation process from Step I to Step V in Figure 8 is repeated until the minimum error ($E_{p}$) is derived. This series of processes is defined as data training, and the fuzzy sets shown in Figure 11 were obtained by means of the data training. Figure 11 shows the modified shape and distribution of fuzzy sets for each input parameter, which are different from those in Figure 10.

## 5. Verification

**Figure 12:**
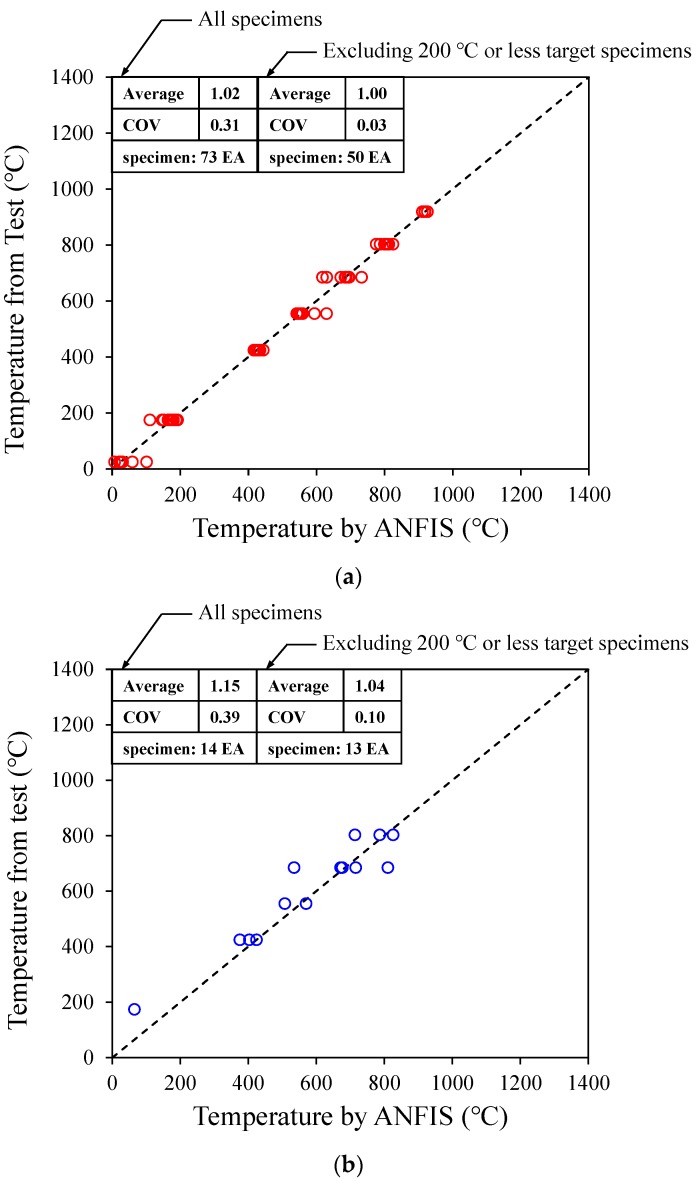
Analysis results of trained and untrained data. (**a**) Trained data; (**b**) Untrained data.

In this study, 73 datasets were randomly extracted from a total of 87 concrete heating test results and used them in the data training of the ANFIS algorithm; the remaining 14 datasets were used for verification. Figure 12 compares the heating temperature evaluated using the proposed ANFIS algorithm and that obtained from the test. Figure 12a shows the analysis results of the 73 datasets used in the training, and Figure 12b shows the analysis results for the remaining 14 datasets, which were not used in the training. As shown in Figure 12a, the average of the ratio of the analysis results to the test results ($T_{test}/T_{ANFIS}$) and COV were 1.02 and 0.31, respectively, with respect to the specimens used in the training. For the specimens excluding the 200 °C or less target specimens, the average of $T_{test}/T_{ANFIS}$ and COV were 1.00 and 0.03, respectively, which confirmed that the training was successful in the ANFIS algorithm. In addition, the heating temperatures of the 14 specimens that were not used in the training by using the proposed ANFIS algorithm were recorded. The results showed that the average of $T_{test}/T_{ANFIS}$ and COV were 1.15 and 0.39, respectively, and the average of the ratio of the analysis results to the test results ($T_{test}/T_{ANFIS}$) and COV were 1.04 and 0.10, respectively, for the specimens excluding the 200 °C or less target specimen. In the case of the target temperature below 200 °C or less, the difference in the heating temperature between the estimation and the experiment was about 100 °C or less. Due to the relatively low target temperature, however, the ratios between the estimated values and the experimental results was about 4 times higher than the ones with the target temperature above 200 °C. In addition, as suggested by the Eurocode 2 [35], the temperature below 200 °C does not significantly affect the concrete member. Thus, the average and COV of the ratios between the estimated values and the experimental results have been provided for two different categories, all specimens and those excluding the ones with the target temperature below 200 °C or less, so that one can compare the accuracy of the estimation in both cases. As a result, it is confirmed that the proposed model provides an accurate heating temperature estimation. If a database having a huge amount of heating temperature data, along with a large number of tests, is built up, the accuracy of the proposed ANFIS algorithm is expected to be further improved.

## 6. Conclusions

In this study, the heating test and non-destructive test on concrete specimens were conducted to identify the heating temperature of fire-damaged concrete. In addition, the ANFIS algorithm for estimating the heating temperature was configured based on the test results, and the accuracy of the proposed model was verified. The following conclusions can be drawn from the findings of this study.

1. The concrete heating test found that spalling occurred at lower temperatures as the concrete compressive strength increased. It was also found that the ultrasonic pulse velocity tended to decrease because of micro-pores and cracks that occurred inside the concrete as the heating temperature rose.
2. The colorimetric analysis revealed that the reflectance of the concrete surface increased as the heating temperature rose and the concrete compressive strength decreased.
3. Based on the test results, this study proposed a heating temperature estimation method using the ANFIS algorithm, in which the ultrasonic pulse velocity, reflectance of the concrete surface, and design compressive strength of the concrete were set as the main input parameters.
4. The heating temperatures of the specimens were evaluated using the proposed ANFIS algorithm. The results showed that the proposed model estimated the heating temperatures of the specimens, which were not used in training, with a high degree of accuracy.
5. In this study, the data training was performed using only the test results of the specimens made of ordinary Portland cement without any admixture. Thus, it is difficult to discuss about the influence of different concrete mixes, for instance, with admixture. Once test data is available, however, the same procedure can be applied to derive the discoloration and heating temperature relationship through the data training process.

## Figures and Tables

**Table 1:** Fire damage rating.

Korea and Japan		US
Grade	Condition	Condition
A	Unaffected	Unaffected
B	Damage to finishing materials. - Soot attached - Heating temperature: below 300 °C	- Normal color - Minor spalling - Non-exposure of rebar (Beam: very minor exposure) - Non-crack and non-deflection
C	No damage to the rebar - Concrete color: Pink - Heating temperature: 300 °C or more - Fine cracks - Fine spalling	- Concrete color: Pink - Localized spalling - Partially exposure of rebar (Column and Beam: up to 25%, non-buckled Wall and Floor: up to 10%, all adhering) - Non-crack and non-deflection
D	Affected on bond performance of main rebar - Cracks on member surface - Partially exposure of rebar	- Concrete color: Light gray - Considerable spalling - Partially exposure of rebar (Column and Beam: up to 50% not more than one rebar buckled wall and floor: up to 20%, generally adhering) - Fine crack - Deflection - (Column: none, other than that: non-significant)
E	Great damage such as main rebar buckling - Great damage on structural members - Exposure of rebar - Great spalling range - Great deformation of structural members	- Concrete color: Light yellow - Almost of surface spalled - Partially exposure of rebar (Column and Beam: up to 50%, more than one rebar buckled, wall and floor: up to 20%, much separated from concrete) - Severe and significant crack - Deflection (Column: any distortion, other than that: severe)

**Table 2:**
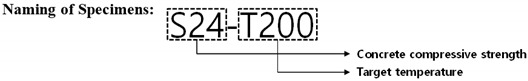
Summary of test specimens.

No.	Concrete Compressive		Number of Specimens	Target Temperature (°C)
	Strength (MPa)			
	Design Strength	Measured Strength		
1	24	25.5	21/each test	25
				200
2	27	28.8		400
3	30	35		500
4	50	50.9		600
5	55	56.9		700
6	80	77.1		800

**Table 3:** Temperature of specimens and furnace at spalling.

Concrete Compressive Strength (MPa)	Target Temperature (°C)	Temperature (°C)	
		Furnace	Specimen
77.1	400	344	268
77.1	500	400	226
		419	239
		420	255
56.9	600	498	310
35.0	700	453	228
56.9	800	453	303
		475	337
Average temperature		433	270

**Table 4:** Ultrasonic pulse velocities of fire-damaged concrete.

Target Temperature (°C)	Ultrasonic Pulse Velocity (km/s)				
	24 MPa * Concrete Specimens	27 MPa * Concrete Specimens	30 MPa * Concrete Specimens	50 MPa * Concrete Specimens	55 MPa * Concrete Specimens
25	5.13	4.17	5.00	4.56	4.92
	4.65	4.15	4.62	4.57	4.65
	4.78	4.21	4.89	4.81	4.72
200	4.41	3.90	4.72	3.96	4.49
	4.21	3.71	4.51	3.98	4.13
	4.45	3.90	4.42	4.07	- **
400	3.08	2.73	3.58	2.56	3.48
	2.82	2.77	3.21	3.10	3.51
	2.96	2.75	3.12	3.01	- **
500	2.41	1.87	2.42	2.21	2.59
	2.12	1.54	2.42	2.06	2.62
	2.42	1.50	2.66	2.10	- **
600	2.14	1.29	2.34	1.39	2.57
	2.10	1.29	2.40	1.43	2.42
	2.10	1.30	2.42	1.31	- **
	1.98	- **	2.36	- **	- **
700	1.55	1.01	1.88	1.05	1.68
	1.52	0.99	1.85	1.12	1.66
	1.51	0.96	1.79	1.04	- **
	1.63	- **	- **	- **	- **
800	- **	0.46	- **	0.92	- **
	- **	- **	- **	0.94	- **
	- **	- **	- **	0.96	- **

* Design compressive strength of concrete. ** Not measurable.

**Table 5:** Concrete discoloration by heating temperature [11,29,30].

Heating Temperature (°C)	Concrete Discoloration
below 300 °C	Sooty
300–600	Pink
600–950	Light gray
950–1200	Light yellow
1200 or more	Concrete melting
